# Murine exposure to gold nanoparticles during early pregnancy promotes abortion by inhibiting ectodermal differentiation

**DOI:** 10.1186/s10020-018-0061-2

**Published:** 2018-12-03

**Authors:** Hui Yang, Libo Du, Guangjun Wu, Zhenyu Wu, Jeffrey A. Keelan

**Affiliations:** 1 Immunology Department, Guang’anmen Hospital, China Academy of Chinese Medical Sciences, Beijing, 100053 China; 2 Institute of Chemistry, Chinese Academy of Sciences, Beijing, 100190 China; 3 Division of Obstetrics & Gynaecology, Faculty of Health and Medical Sciences, University of Western Australia, Perth, WA Australia

**Keywords:** Gold nanoparticles, Pregnancy, Abortion, Materno-fetal transport, Ectodermal differentiation

## Abstract

**Background:**

Gold nanoparticles (AuNPs) have been widely studied for biomedical applications, although their safety and potential toxicity in pregnancy remains unknown. The aim of this study is to explore the effect of AuNPs maternal exposure at different gestational ages on fetal survival and development, as well as the potential mechanism of AuNPs affecting embryos and fetuses.

**Methods:**

Thirty nm polyethylene glycol (PEG)-coated AuNPs (A30) were administered to pregnant mice via intravenous injection (5 μg Au/g body weight) over three days at either early or late pregnancy. Fetal abortion rate and morphological development in E16.5 were then detected in detail. The pregnant mice physiological states with A30 exposure were examined by biochemical, histological or imaging methods; and materno-fetal distribution of gold elements was assayed by electron microscopy and mass spectrometry. Murine embryonic stem cells derived embryoid-bodies or neuroectodermal cells were treated with A30 (0.0025 to 0.25 μg Au/mL) to examine A30 effects on expression levels of the germ differentiation marker genes. Tukey’s method was used for statistical analysis.

**Results:**

Exposure to A30 during early (A30E) but not late (A30L) pregnancy caused a high abortion rate (53.5%), lower fetal survival rate and abnormal decidualization compared with non-exposed counterparts. The developmental damage caused by A30 followed an “all-or-nothing” pattern, as the non-aborted fetuses developed normally and pregnancies maintained normal endocrine values. A30 caused minor impairment of liver and kidney function of A30E but not A30L mice. TEM imaging of fetal tissue sections confirmed the transfer of A30 into fetal brain and live as aggregates. qPCR assays showed A30 suppressed the expression of ectodermal, but not mesodermal and endodermal differentiation markers.

**Conclusions:**

These results illustrate that maternal A30 exposure in early pregnant results in A30 transfer into embryonic tissues, inhibiting ectodermal differentiation of embryonic stem cells, leading to abnormal embryonic development and abortion. While exposure to A30 during late pregnancy had little or no impact on dams and fetuses. These findings suggest the safety of biomedical applications employing AuNPs during pregnancy is strongly influenced by fetal maturity and gestational age at exposure and provide the clues for AuNPs safe application period in pregnancy.

**Electronic supplementary material:**

The online version of this article (10.1186/s10020-018-0061-2) contains supplementary material, which is available to authorized users.

## Highlights

Murine exposure to 30 nm PEGylated AuNPs (A30) during early pregnancy, but not late pregnancy, promotes abortion.

The developmental damage caused by A30 followed an “all-or-nothing” pattern.

Exposure to A30 has little or no negative effect on maternal physiological status.

A30 placental transfer into the fetal organs results in aggregates in membrane vesicles.

A30 suppress neuroectodermal differentiation of murine embryonic stem cells R1, which could be the main mechanism of A30-induced fetal injury and abortion.

## Introduction

With the rapid development of nanotechnology, a number of nanoparticles (NPs) have been approved for use in the medical, pharmaceutical and cosmetic fields (Bowman et al., 2010; Petros & DeSimone, 2010; Yang et al., 2012; Tian et al., 2013). Due to their unique electronic and optical features, gold nanoparticles (AuNPs) have been used for biomarker detection, drug and gene delivery, molecular imaging and photothermal/photodynamic treatment (Boisselier & Astruc, 2009; Chen et al., 2007; Huang et al., 2007; Podsiadlo et al., 2008). Accordingly, substantial attention has been paid to the biological effects, toxicity and pharmacokinetics of AuNPs and their interactions with proteins and cells (Świdwińska-Gajewska & Czerczak, 2017; Feliu et al., 2016; Hornos Carneiro & Barbosa Jr., 2016; Wang et al., 2015; Araújo et al., 2015). Exposure to nanoparticles in pregnancy carries additional risks due to the potential for fetal exposure and teratogenesis or developmental distrurbances; hence, there are growing concerns regarding potential hazards resulting from exposure to nanosized materials in pregnancy (Li et al., 2014; Ema et al., 2010). However, the potential adverse effects of AuNPs on pregnancy and embryonic development are not thoroughly understood.

Previous studies have addressed the transplacental transport characteristics of AuNPs, including the window of time for transportation, the influence of surface modification and scale of AuNPs on transportation, and the metabolism and distribution of AuNPs throughout the body during pregnancy (Yang et al., 2012; Tian et al., 2013; Yang et al., 2014). Data on the genetic and developmental toxicity of AuNPs mainly come from zebrafish and in vitro cell culture research. Some recent studies have reported that AuNPs might induce DNA damage and chromosomal abnormalities of both human and murine cells (Xia et al., 2017; Di Bucchianico et al., 2014), while another report showed that AuNPs of 1.4 nm could bind to DNA directly (Pan et al., 2007). Exposure of AuNPs with different functional groups were reported to cause mortality, developmental disorders, hypolocomotor activity and abnormal behavioural activity of zebrafish embryos (Kim et al., 2013; Harper et al., 2011; Truong et al., 2012). However, whether these in vitro studies could be extrapolated to the human situation remained uncertain, due in part to the significant impact of comparative placentation on the extent of fetal exposure (Aillon et al., 2009).

Murine pregnancy models are often used to study how xenobiotics delivered to the mother may affect fetal development, because the rodent placenta has some similarities in structure and transport properties to that of the human (Georgiades et al., 2002). Studies on the effects of AuNPs on the development of fetal mice are limited, however. Shmada et al. found there was no toxic damage to the placentas and fetal organs of the mice treated with 20 and 50 nm GNPs on gestation day 16 (GD16) and GD17, although AuNPs were reported to be visualised in fetal endothelial cells (Rattanapinyopituk et al., 2014). Balansky et al. found that AuNPs of 100 nm (but not 40 nm) could induce chromosomal damage of exposed fetal mice on GD 10, 12, 14, and 17 (Balansky et al., 2013). At present, most studies on the effects of NP on murine fetuses focus on the late pregnancy fetus, with little attention paid to embryo in earlier pregnancy.

The sensitivity of mammalian embryos to xenobiotics varies at different gestational ages. Regarding the response of human fetus to drugs in maternal circulation, the embryos/fetuses are considered safe within first 3 weeks, highly sensitive from 5 to 8 weeks, moderately sensitive from 8 to 20 weeks, and hyposensitive after 20 weeks (Buhimschi & Weiner, 2009). Whether the effects of AuNPs on the fetus follow the similar response pattern as the drugs remains unknown. Any such differences are likely to be due to significant differences in materno-fetal physiology and anatomy at different stages of pregnancy, such as the structure and function of the placenta, extent of maternal-to-fetal transmission and the status of fetal organ development (Krantz et al., 1962). Accordingly, the effects of exposure to NPs on fetal development at different gestational ages are likely to be different, which would have important implications for the safety and efficacy of biomedical applications of NPs during pregnancy.

A systematic study of the impact of NPs, including AuNPs, on fetal development at different stages of mammalian pregnancy has not yet been carried out. Most studies regarding developmental safety of NPs are focused on mid-term and late pregnancy (Świdwińska-Gajewska & Czerczak, 2017; Rattanapinyopituk et al., 2014; Balansky et al., 2013), namely, the second and third trimester of human pregnancy. Our previous studies found that tail vein injection of AuNPs (0.9 μg Au/g body weight) to pregnant mice from GD5.5 to GD13.5 did not affect fetal survival and offspring health (Yang et al., 2012). The dose used in the above study is about 0.025% of the LD50 of AuNPs in mice (Hainfeld et al., 2006), which is much lower than the dose for medical use (McMahon et al., 2008). However, at the higher dose of  4.5 μg Au/g body weight, 30 nm AuNPs could cause perceptible maternal alveolar damage at GD9.5 (Yang et al., 2012), while three consecutive doses of 5 μg Au/g body weight resulted in transient renal injury in female mice (data not published). Therefore, it is critical to evaluate the effect of AuNPs on embryonic development on different gestational ages at a clinically relevant dose and/or under repeated exposure conditions. Moreover, there is a need to discern whether the potential effect of AuNPs is exerted directly on the fetus or indirectly via changes in maternal physiological status. Such studies are vital in understanding how systemically-delivered AuNPs interact embryonic developmental process (Hougaard et al., 2015; Sun et al., 2013).

In the present study, we assessed how the embryo and fetal development are influenced when maternal mice are exposed to 30 nm polyethylene glycol (PEG)-coated AuNPs (A30) in early and late pregnancy. Our study found that maternal exposure to AuNPs in maternal circulation in early murine pregnancy can damage embryo development leading to abortion, potentially via a mechanism involving DNA damage.

## Materials and methods

### Synthesis and characterization of 30 nm polyethylene glycol (PEG)-coated AuNPs (A30)

A30 (Additional file 1: Figure S1A and B) were prepared by the citrate reduction method combined with PEG replacement according to published methods (Yang et al., 2012). The morphology and size of *A30* were measured using transmission electron microscopy (TEM, JEM-200CX, Jeol Ltd., Japan) (Additional file 1: Figure S1C). The nanoparticles’ surface charge (zeta potential, mV) was assessed using a Zetasizer Nano series Nano ZS (Malvern Instruments Ltd., Malvern, UK) as − 3.0 mV in 0.9% saline. All A30 suspensions were sonicated for 5 min before use.

### Exposure to A30 and animal sampling

**Fig. 1:**
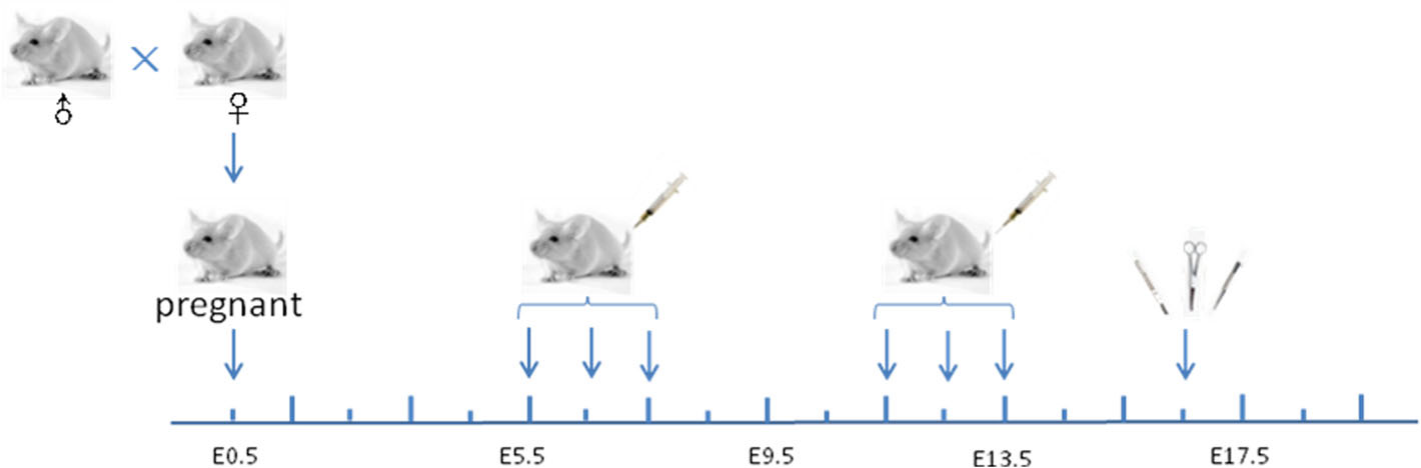
Schematic illustration of A30 exposure and animal processing and sampling. CD-1® (ICR) mice were mated overnight and the presence of a vaginal plug on female mice was designated as embryonic day 0.5 (E0.5). For early pregnancy exposure, daily intravenous injection was started from E5.5 for 3 consecutive days, with the injection vehicle and dose as described in the Methods; for late pregnancy exposure, daily intravenous injections commenced on E11.5 and continued for 2 more days. Various test samples from maternal and fetal tissues were collected at E16.5

All animal procedures were approved by the ethics committee of Guang’anmen Hospital, China Academy of Chinese Medical Sciences and were performed as described previously (Yang et al., 2012). Female CD-1®(ICR) mice at 8–10 weeks of age and between 25 and 30 g were obtained from Vital River Inc. (Beijing, China). A schematic of the experimental process is illustrated in Fig. 1. The mice were mated overnight and the presence of a vaginal plug on female mice was designated as a pregnant mouse at embryonic day 0.5 (E0.5). The pregnant mice were randomly divided into 5 groups as follows. (1) NP group: Normal Pregnant mice receiving no treatment. (2) The A30E group: pregnant mice that received daily tail vein injection of 100 ul A30 suspension during early pregnancy on E5.5, E6.5 and E7.5 consecutively, the dose of A30 is 5 μg Au/g body weight. (3) The NE group: pregnant mice received 100 μL of 0.9% saline injection during early pregnancy at E5.5, E6.5 and E7.5 consecutively as control. (4) The A30L group: pregnant mice that received A30 tail vein injection (same dose as above) during late pregnancy on E11.5, E12.5 and E13.5 consecutively. (5) The NL group: pregnant mice received 100 μL of 0.9% saline injection during late pregnancy at E11.5, E12.5 and E13.5. The weight of pregnant mice was recorded every other day until E16.5 when all mice were sacrificed by exsanguination under anesthesia. The number of live fetuses and the number of stillbirths in each pregnant mouse were counted and recorded. Uteri without fetal content were identified as aborted, and turbid red or black contents in the uterus were identified as stillbirths. Maternal blood serum, maternal organs and tissues, intact fetuses and fetal organs were collected separately for the subsequent pathological examination or embryo development analysis.

### Endocrine and hematological assessments in pregnancy

Routine hematological examination was performed on blood from E16.5 pregnant mice following addition of 20 u/ml sodium heparin using an Automatic Hematology Analyzer (Sysmex Xn-550, Japan) to observe the number and distribution of blood cells. Serum was also collected from maternal blood at E16.5 following centrifugation. Serum liver and kidney function indexes (including alkaline phosphatase, creatinine, urea nitrogen) and hormonal status (17‧-estradiol and progesterone concentrations) were assessed using an automated clinical chemistry analyzer (Olympus Au640, Japan).

### Fetal organ and bone development

Two or three fetuses from each litter were fixed in Bouin’s solution (Sigma-Aldrich, U.S.A.) for a week, then the size and shape of fetal organs were observed under a stereomicroscope (Motic, China). Two or three fetuses from the same litter were fixed in 95% ethanol solution for 48 h, then transferred to 1% potassium hydroxide for 48 h. After that, the internal organs and fat of these fetuses were removed and the skeletons were put into 1% potassium hydroxide until the bones were clear. Alizarin red solution at 2% was used to dye the bones for evaluation of bone deficiency.

### Histological examination

Maternal liver, maternal kidney and fetuses were fixed in 10% neutral buffered formalin solution overnight, embedded in paraffin blocks, then sectioned at 2 mm on a Leica CM1850 microtome. Bright-field images of haematoxylin-eosin (H&E) or periodic acid-Schiff (PAS) stained sections were acquired on a Leica DM1-3000B microscope equipped with a Nikon DXM1200 color CCD camera (Nikon Instruments Inc., Melville, NY). Image-Pro Plus image processing and analysis software (MediaCybernetics, U.S.A.) were used for image capture.

### ICP-MS analysis for gold element content in tissues

At E16.5, the maternal liver, lung, kidney, placenta, amniotic fluid and blood, fetal liver and brain tissues were collected and weighed, then digested in *aqua fortis* containing nitric acid and hydrochloric acid at a ratio of 3:1. After adjusting the solution volume to 5 ml using 2% nitric acid and 1% hydrochloride acid (1:1), gold concentrations were determined by quantitative inductively coupled plasma mass spectrometry (ICP-MS) using an ELAN DRC-e ICP-MS instrument (Perkin Elmer, Massachusetts, USA). The limit of quantification was 10 ng/ml.

### Transmission electron microscope (TEM) analysis of tissue sections

At E16.5, the fetal liver and brain were excised and fixed in 2.5% glutaraldehyde for 2 h. Small pieces of tissues collected from these samples were fixed in 1.5% osmium tetroxide in sodium cacodylate buffer for 60 min at 4 °C. After dehydrated and propylene epoxide treatment, samples were embedded in Epon resin and polymerized sequentially at 40 °C(2 h), 60 °C(4 h) and 80 °C(10 h). Ultrathin sections were prepared with ULTRACUT UCT/UC6 (Leica, Germany). After double staining with uranium acetate and lead citrate, sections were observed and imaged under a Tecnai Spirit transmission electron microscope (FEI, U.S.A.).

### Scanning electron microscopy (SEM) analysis of the decidua

Decidual tissue with diameter of 1 mm from normal pregnant mice in the NP group and aborted mice of the A30E group were fixed in 2.5% glutaraldehyde for 2 h, then fixed in 1% osmic acid at 4 °C for 1 h. After washed with 0.1 M PBS, tissue samples were in turn dehydrated and with 50% ethanol, 70% ethanol, 90% ethanol, 100% ethanol (3 times), then ethanol:isoamyl acetate (1:1), and 100% isoamyl acetate. Samples were dried on a Samdri®-795 ‍Critical point drier (Tousimis, U.S.A.) and sprayed with 10 nm metal conductive thin layer on an EMS 550 Ion beam sputtering coating instrument (EMS, U.S.A.). Samples were then observed and imaged under scanning electron microscope Teneo SEM (FEI, U.S.A).

### Murine embryonic stem cells R1 (MESC-R1) culture and embryoid body formation

MESC-R1 cells were cultured under typical embryonic stem (ES) cell condition in DMEM media supplemented with 15% fetal calf serum (Hyclone), LIF (Chemicon), non-essential amino acids (Gibco), GlutaMax-I (Gibco), β-mercaptoethanol (Gibco) and penicillin/streptomycin at 37 °C, under 5% CO_2_. For feeder-free culture, 2 × 10^5^ MESC-R1 cells were plated on gelatinized 6-well tissue-culture plates (Corning Costar, NY) and Knockout Serum Replacement (KOSR) (Gibco) was used to substitute fetal calf serum. For embryoid bodies (EBs) formation, monolayer undifferentiated MESC-R1 cells at 90% confluence were trypsinized into single cells and then replaced at a density of 3 × 10^5^ cells per 60 mm non-adherent dish in KOSR-containing ES cell medium devoid of LIF. The remaining trypsinized undifferentiated MESC-R1 cells were collected as self-renewal control. A30 was added into the medium of EBs formation at a final concentration of 0.25 μg Au/ml the addition time point was defined as day 0. Floating EBs were harvested at day 6 by centrifugation at 3000 rpm/min for 10 min and processed for mRNA expression analysis of germ layer differentiation markers.

### Ectodermal differentiation and A30 treatment of MESC-R1 cells

MESC-R1 cells growing on feeder cells were adapted to culture on 0.1% gelatin-coated plastic dishes for 2–3 passages to eliminate feeder cells. MESC-R1 cells were then trypsinized and plated onto gelatin-coated plastic at a density of 0.5–1.5 × 10^4^/cm^2^ in KOSR ES medium without LIF. After 24 h, the medium was changed to N2/B27 medium with beta-mercaptoethanol (Invitrogen Gibco) and Glutamax-I (Invitrogen Gibco). This time was recorded as day 0 of differentiation. At the same time, A30 was added into the medium at a series of final concentrations from 0.0025 to 0.25 μg Au /ml. MESC-R1 cells were harvested by centrifugation at 3000 rpm/min for 10 min at day 5 to analyze the expression of ectodermal differentiation markers Sox1, Sox3 and Tubb3.

### Quantitative real-time PCR analysis of expression of germ layer differentiation markers

Total cellular RNA was isolated with Trizol (Invitrogen, U.S.A.). The oligo dT-primed first-strand cDNA was synthesized using reverse transcriptase (Toyobo, Japan). Primers sequences for the germ layer differentiation markers are listed in Table 1. The quantitative real-time PCR (qPCR) assay was carried out on a Mx3000P detection system (Stratagene, U.S.A) using EvaGreen dye (Biotium, U.S.A). qPCR process: pre-incubation, 95 °C for 10 min; amplification, 95 °C for 10s, 59 °C for 60s, 40 cycles; melting curves, 95 °C for 15 s, 72 °C for 15 s, 95 °C for 15 s. The ΔΔCt method was used to comparatively quantify the amount of mRNA levels.

**Table 1:** Primer sequences of the germ layer differentiation markers for qPCR assay

Primer sequence (5’—3’)	Primer name	Purification method	Attribution of differentiation marker
CATGGCCTTCCGTGTTCCTA	mGAPDH_F	PAG	Internal reference
CCTGCTTCACCACCTTCTTGAT	mGAPDH_R	PAG	Internal reference
ACCTGAGCTATAAGCAGGTTAAGAC	mNanog_F	PAG	Sternness
GTGCTGAGCCCTTCTGAATCAGAC	mNanog_R	PAG	Sternness
TTACTTCCCGCCAGCTCTTC	rnSoxl_F	PAG	Neuroectederm
TGATGCATTTTGGGGGTATCTCTC	mSoxl_R	PAG	Neuroectoderm
GGCAACTATGTAGGGGACTCAG	mTubb3_F	PAG	Neuroectederm
CCTGGGCACATACTTGTGAG	mTubb3_R	PAG	Neuroectederm
CTCCAACCTATGCGGACAAT	mT_F	PAG	Mesendoderm
AGACTGGGATACTGGCTAGAG	mT_R	PAG	Mesendodetm
CCCATCCTGGACCGTTTCC	mLhxl_F	PAG	Mesoderm
CGCTTGGAGAGATGCCCTG	mLtaxl_R	PAG	Mesoderm
CACATGAAGGAGTACCCGGACTA	mSox3_F	PAG	Ncurocctodcrm
TGAGCAGCGTCTTGGTCTTG	mSox3_R	PAG	Neuroectederm
CGAGCCAAAGCGGAGTCTC	mSoxl7_F	PAG	Fmlodenri
TGCCAAGGTCAACGCCTTC	mSoxl7_R	PAG	Fndodcrm

### Ammonium sulfide staining

At E16.5, uteri with no fetuses were excised longitudinally and fixed in 10% ammonium sulfide for 20 min to determine the presence and number of implantation sites.

### Statistical analysis

All results are presented as mean ± standard deviation (s.d.). Statistical significance of the differences between groups was evaluated by Tukey’s method after analysis of variance (ANOVA).

## Results and discussions

### The abortion rate of pregnant mice exposed to AuNPs in early pregnancy was significantly higher than that of non- exposed pregnant mice

**Fig. 2:**
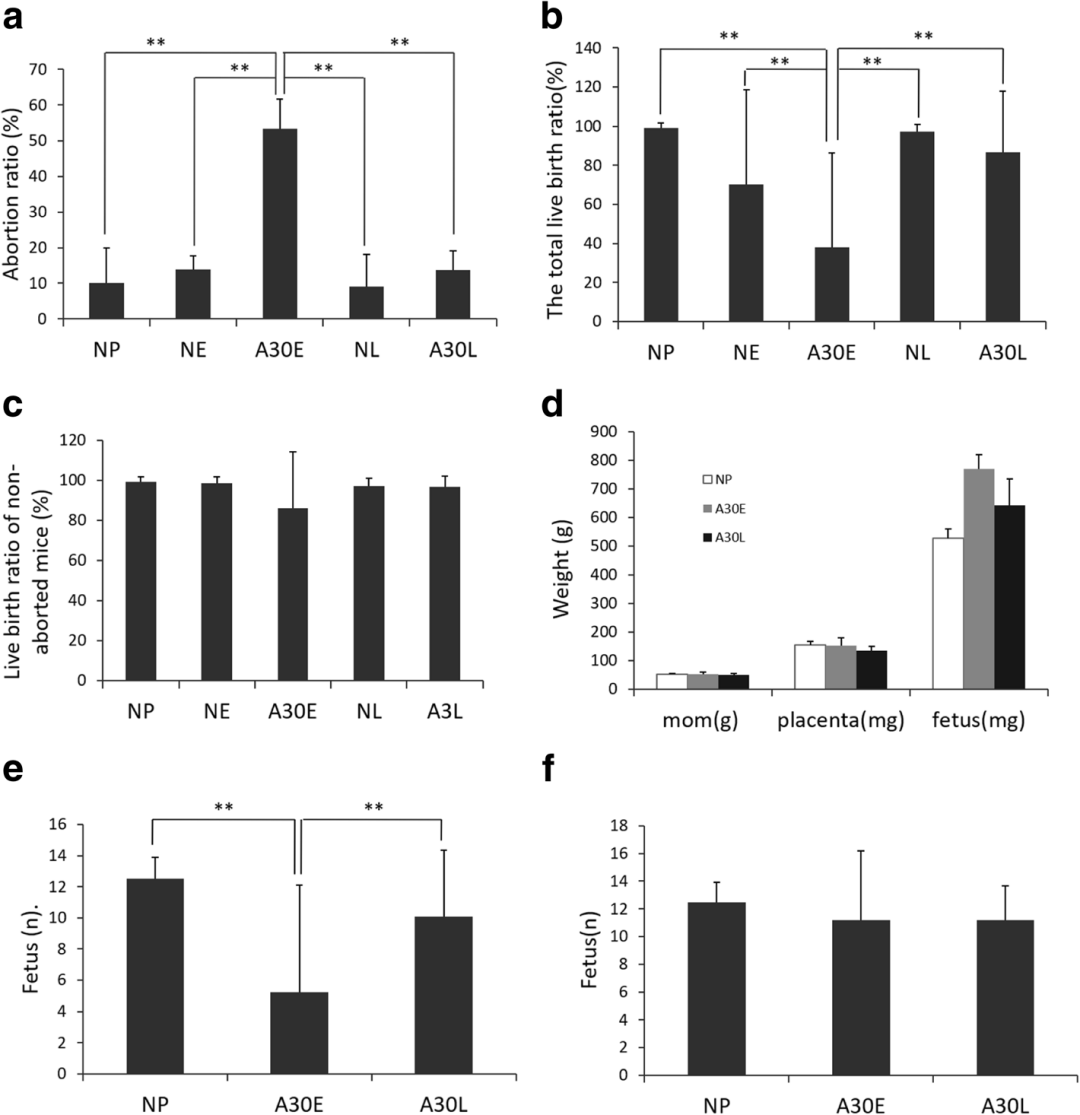
Effects of A30 exposure at different gestational ages on abortion rate and fetal survival. **a** Abortion rate is the ratio of the number of aborted mice to the total number of pregnant mice in a group (*n* = 10 per group, three independent experiments). **b** Total live birth rate is the ratio of the surviving embryo to the total embryos of a pregnant mice (n = 10 per group). **c** Live birth rate in the non-aborted mice is the ratio of the surviving embryos to the total embryos in a non-aborted pregnant mouse (*n* = 5–10 per group). **d** The mean of maternal, placental and fetal weights of non-aborted pregnant mice at E16.5 (*n* = 5–10 per group). **e** The mean number of fetuses per dam (*n* = 10 per group). **f** The mean number of viable fetuses in non-aborted pregnant mice (*n* = 5–10 per group). Data are shown as mean ± s.d. The symbol ** represents *p* < 0.01 by ANOVA with Tukey’s test post-hoc

A previous study demonstrated that gestational age is an important factor that influences the consequence of fetal exposure to NPs (Yang et al., 2012). In this study, A30 was administered via intravenous injection (i.v.) to pregnant mice over three days in the early or late pregnancy period to determine the effect of exposure to AuNPs on fetal development at different gestational ages. The pregnant mice were executed on E16.5 and the uterus and fetal development were examined after opening the maternal abdominal cavity. Normal pregnancy uteri (non-aborted) showed a continuous ‘chain of beads’, with a live fetus in each “bead”; nothing in the uterus (empty uterus) was identified as abortion; of course, there were very few non-aborted uteri involving a single abortion site or stillbirth (Additional file 1: Figure S2). The abortion rate of A30E group was 53.5 ± 8.1%, significantly higher than that of A30L group (13.7 ± 5.5%) and unexposed control groups (NE, saline i.v. injection in early pregnancy, 13.9 ± 3.8%; NL, saline i.v. injection in late pregnancy, 9.1 ± 9.1%; and NP, pregnant mice received no treatment, 10.0 ± 10.0%) (*p* < 0.01) (Fig. 2a). Correspondingly, A30E mice exhibited lower live birth rate (38.1%) (Fig. 2b) and lower mean fetus number per litter (Fig. 2e) compared with the A30L group and A30 unexposed control groups (*p* < 0 .01). For the A30-treated non-aborted pregnant mice, there was no significant difference in the live birth rate (Fig. 2c) or mean fetus number per litter (Fig. 2f) between A30E and A30L groups or between A30E and NP groups (*p* > 0.05). At the E16.5 checkpoint, the maternal and placental weight of non-aborted pregnant mice in each group were similar (Fig. 2d). These results indicate that A30 exposure at early pregnancy of mice (A30E) led to a significantly higher incidence of abortion. However, fetal development of non-aborted mice in each group was not significantly affected even when exposed to A30 during early pregnancy (A30E), suggesting that only certain dams are susceptible to fetal loss.

Ammonium sulfide staining of aborted mice uteri in A30E and A30L groups confirmed the presence of implantation sites on the inner wall of uterus without embryo at E16.5 (Additional file 1: Figure S3), indicating that implantation had occurred in these uteri before a miscarriage due to exposure to A30. We monitored the weight changes of each pregnant mouse in detail to determine the exact time pointfor implantation number being affected after A30 treatment (Fig. 4a). During the period from E0 to E7.5, all pregnant mice in NP, A30E and A30L groups gained a slow but sustained increase in body weight, while the non-pregnant mice in NN group did not grow in weight. After E9.5, most mice in A30L and NP groups began to grow rapidly, but a significant proportion of mice in A30E stopped gaining weight (Fig. 4a), which was consistent with the high abortion rate in this group (Fig. 2a). This suggests that the implanted embryos were aborted during E8.5- E9.5. All pregnant mice that failed to exhibit significant weight gains after E9.5 were shown, using laparotomy at E16.5, to have aborted.

### The development of non-aborted fetuses

**Fig. 3:**
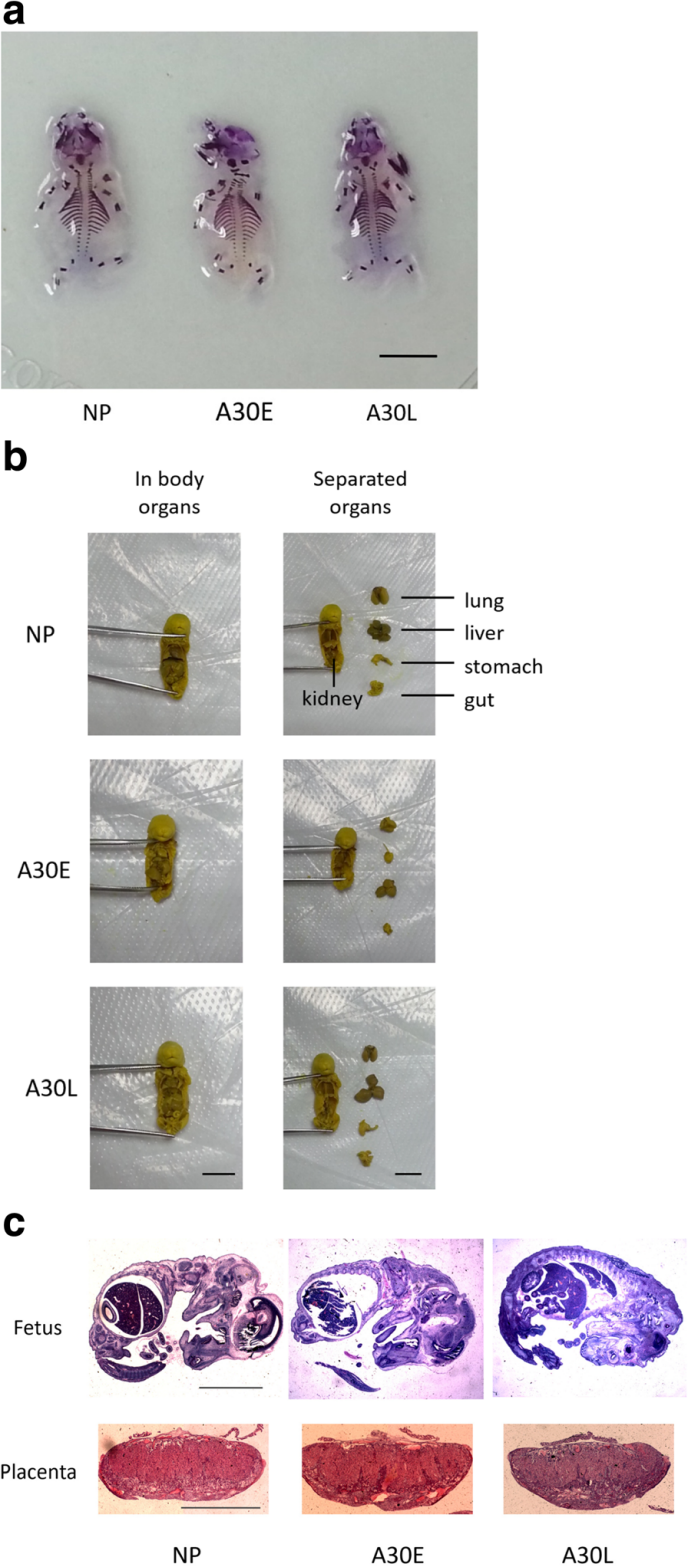
Development of non-aborted, viable fetuses in each group. **a** Skeletal development of E16.5 fetuses. Scale bar is 1 cm. **b** Visceral development of E16.5 fetuses. Scale bar is 1 cm. **c** H&E staining of E16.5 fetuses and PAS staining of E16.5 placental tissues. Representative images of at least 4 samples for each group are shown. Scale bar are 1 cm (fetus) and 0.5 cm (placenta)

Although A30 exposure in early pregnancy resulted in miscarriage (53.5% abortion rate), there were still some non-aborted fetuses in A30E group. The placentas and viable fetuses in the A30E group showed a similar shape, color and quantity to those of NP and A30L fetuses (Additional file 1: Figure S4). The skeletons of viable fetuses at E16.5 were stained with Alizarin Red; it was found that, for all the tested fetuses from NP, A30E and A30L groups, the cranial bones exhibited normal shape and gaps, the vertebrae showed structural integrity, the scapula was symmetrical and the long bones were normal (Fig. 3a). The fetal organs were fixed in situ with Bouin’s solution to facilitate observation of their shape and position. The fetal organs of the A30E and A30L groups were fully developed and no morphological and positional abnormalities were observed compared with those of NP group (Fig. 3b). We also analyzed the placental layer structure of mice in each group by periodic acid-Schiff (PAS) staining and found that there were no significant differences in labyrinth layer thickness or morphology between NP and A30-exposed mice (Fig. 3c), suggesting that exposure to A30 during pregnancy did not damage the normal placental structure which is important for fetal growth and development .

Although exposure to A30 during early pregnancy significantly increased the murine abortion rate (Fig. 2), there was still a certain proportion of normal pregnancies and normally developing fetuses under the same early pregnant exposure condition (Fig. 3 and Additional file 1: Figure S4). This phenomenon is consistent with an “all-or-nothing” pattern, where there is either no effect or considerable effect of exposure on fetal losses. For late pregnant mice, maternal A30 exposure did not increase the abortion rate nor cause fetal malformation (Fig.2a and Fig.3). Just as human embryos have different teratogenic sensitivity to exogenous substances and drugs at different gestational ages (Sun et al., 2013), fetotoxicity of A30 may also be gestational age-dependent.

### Maternal physiological changes under exposure to A30 at early or late pregnancy

**Fig. 4:**
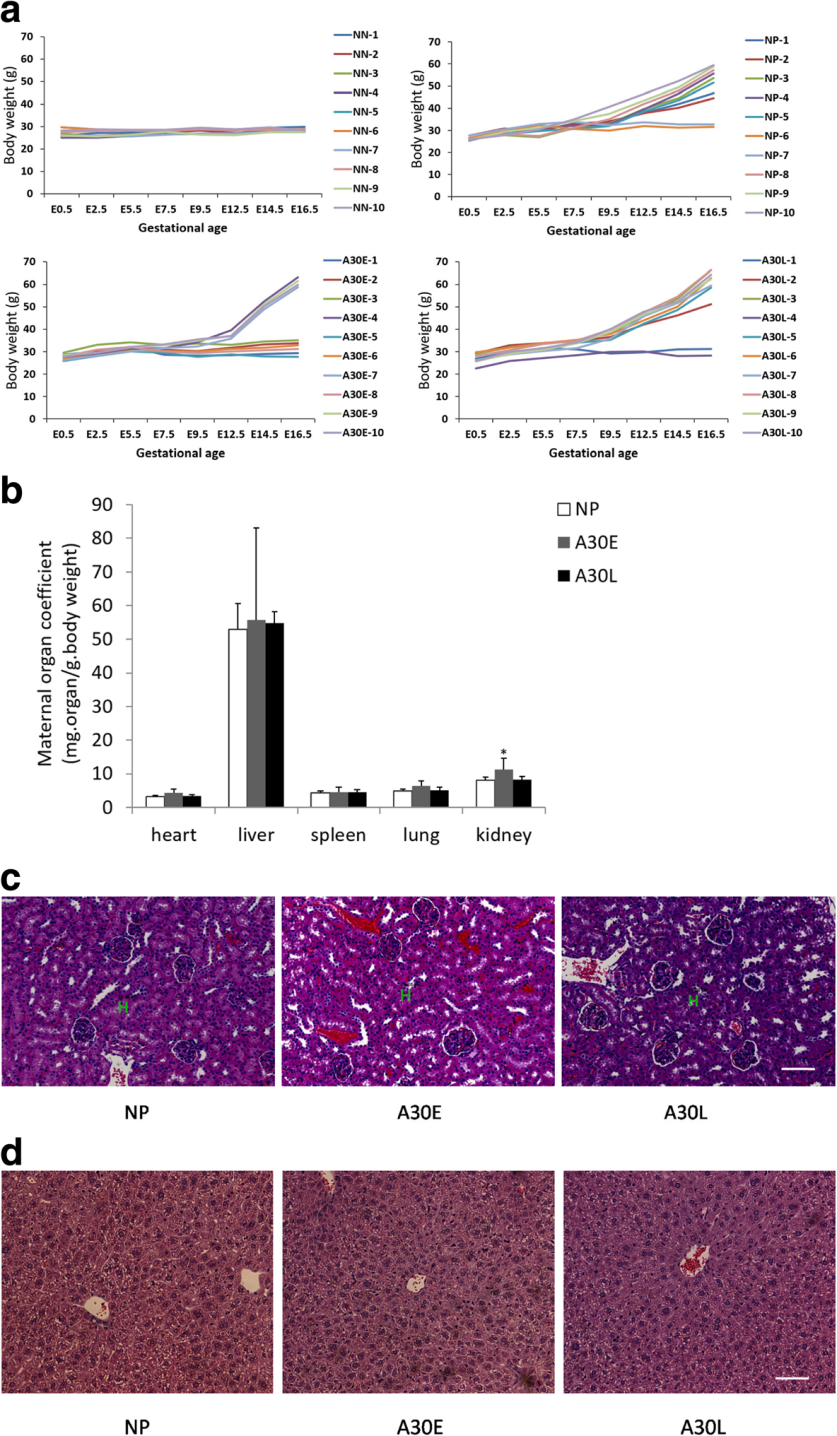
Maternal physiological changes under exposure to A30 during early or late pregnancy. **a** The rate of increased weight of pregnant mice. NN is defined as normal non-pregnant mice. **b** Organ coefficient of pregnant mice. Data are shown as mean ± s.d. (n = 10 per group). The symbol * represents *p* < 0.05 compared with NP group by ANOVA with Tukey’s test post-hoc. **c** H&E staining of maternal kidney of pregnant mice. **d** H&E staining of maternal liver of pregnant mice. For (**c**) and (**d**), representative images of at least 4 samples for each group are shown. The scale bar is 50 μM

Maternal changes caused by exposure to exogenous substances during pregnancy might affect the fetuses in many ways, either directly or indirectly (Plotka et al., 2014). In the present study, as seen from Fig. 4b, the maternal kidney organ coefficient in the A30E group (but not A30L group) slightly increased compared with that of the NP group (*p* < 0.05). H&E staining on kidney sections showed no obvious difference on glomerular distribution density and glomerular morphology between A30-exposed and NP animals (Fig. 4c), Correspondingly, blood creatinine and urea nitrogen concentration did not increase in either the A30E or A30L groups compared with the NP group (Table 2), indicating normal glomerular filtration rates in both A30E and A30L groups. H&E staining of liver sections showed no change in liver morphology and structure after A30 exposure (Fig. 4d). The concentration of ALP, but neither ALT nor AST, increased in the A30E group compared with that in NP group (Table 2), suggesting there was a certain degree of hepatobiliary duct obstruction in the pregnant mice of the A30E group. Our previous studies have shown that PEG-coated AuNPs have very limited influence on the physiological status of pregnant mice (Yang et al., 2012).

**Table 2:** Biochemical analysis of blood serum of pregnant mice

	ALT (IU/L)	AST (IU/L)	ALP (IU/L)	TP (g/L)	ALB (g/L)	A/G	BUN (mmol/L)	Cr (μmol/L)
NP	30.7 ± 8.6	120.4 ± 20.5	74.9 ± 12.4	45.9 ± 3.2	29.1 ± 1.8	1.8 ± 0.3	10.0 ± 1.3	16.0 ± 1.6
A30E	35.1 ± 7.2	113.1 ± 7.5	101.3 ± 45.2*	52.7 ± 7.1	34.1 ± 6.4	1.9 ± 0.4	7.0 ± 1.0*	12.4 ± 2.0*
A30L	28.9 ± 4.9	107.6 ± 13.6	70 ± 17.1	47.9 ± 4.4	28.9 ± 2.8	1.5 ± 0.1	10.7 ± 1.4	15.9 ± 2.1

*ALT* anine aminotransferase, *AST* aspartateaminotransferase, *ALP* Alkaline phos- phatase, *TP* Total Protein, *ALB* albumin, *A/G* Albumin globulin ratio, *BUN* usea nitrogen, *Cr* creatinine. *p < 0.05 compared with NP group by ANOVA with Tukey’s test post-hoc. *n* = 7–10 per group

Red blood cell count, hemoglobin and hematocrit were all reduced by approximately 10% in the A30E and A30L groups compared with the NP animals (Table 3a). However, the mean corpuscular volume and the mean concentration of hemoglobin were not changed, suggesting that A30 exposure had little if any effect on functional oxygen transport. The absolute number and percentage of basophils in white blood cells in the A30E and A30L dams were significantly higher than in the NP group (Table 3b). In the A30E group, platelet count increased while the proportion of large-type platelets decreased compared with A30L and NP groups (Table 3c). Therefore, exposure to A30 during pregnancy may result in a maternal allergic-type response. Whether or not these changes are related to abortion is uncertain.

**Table 3:** Hematological assessment of pregnant mice

(a) Red blood cell count and analysis											
	RBC (× 10^12^/L)		HGB (g/L)		HCT (%)		MCV (fL)	MCH (pg)	MCHC (g/L)	RDW (%)	
NP	9.2 ± 0.6		145.7 ± 10.4		42.8 ± 2.8		46.5 ± 1.6	15.9 ± 0.6	340.3 ± 4.8	21.7 ± 1.2	
A30E	8.1 ± 0.7*		125.1 ± 11.2*		37.7 ± 2.7*		46.9 ± 2.3	15.5 ± 0.6	331.9 ± 7.6	22.6 ± 2.1	
A30L	8.2 ± 0.7*		126.6 ± 9.9*		38.2 ± 2.4*		46.9 ± 2.2	15.5 ± 0.5	331.3 ± 10.8	21.3 ± 1.2	
(b) White blood cell count and analysis											
	WBC	LYM	LYM	MON	MON	NEU	NEU	EOS	EOS	BAS	BAS
	(× 10^9^/L)	(× 10^9^/L)	(%)	(× 10^9^/L)	(%)	(× 10^9^/L)	(%)	(× 10^9^/L)	(%)	(× 10^9^/L)	(%)
NP	3.8 ± 0.8	3.2 ± 0.6	86.3 ± 6.1	0,1 ± 0.04	1.7 ± 1.2	0.2 ± 0.1	3.6 ± 2.2	0 ± 0	0 ± 0	0.3 ± 0.2	8.7 ± 4.3
A30E	6.3 ± 2.2*	4.6 ± 1.9	79.5 ± 5.7	0.1 ± 0.06	1.7 ± 0.8	0.2 ± 0.06	2.9 ± 0.3	0 ± 0.02	0.2 ± 0.4	1 ± 0.7*	14.4 ± 5.3*
A30L	8.6 ± 1.9*	6.5 ± 1.6*	78.1 ± 6.4	0.2 ± 0.1	2.3 ± 0.9	0.5 ± 0.1*	6.3 ± 1.4*	0.1 ± 0.1	1.1 ± 1.2	1.1 ± 0.4*	13.1 ± 6*
(c) Platelet analysis											
	PLT (× 10^9^/L)		RDW-CV (%)		PDW (%)		MPV (fL)	P-LCR (%)		PCT (%)	
NP	583.6 ± 294.3		21.7 ± 1.2		8 ± 1.7		7.4 ± 0.7	9.6 ± 5.2		0.5 ± 0.2	
A30E	804.6 ± 127.3*		22.6 ± 2.1		7.5 ± 0.4		7.1 ± 0.3	6.6 ± 1.2*		0.6 ± 0.1	
A30L	476.4 ± 293.5		21.3 ± 1.1		8.8 ± 0.7		8 ± 0.4	10.6 ± 2.7		0.4 ± 0.2	

(a) Red blood cell count and analysis. *RBC* red blood cell count, *HGB* hemoglobin, *HCT* red blood cell specific volume, *MCV* mean cell volume, *MCH* mean corpsular hemoglobin, *MCHC* mean corpuscular hemoglobin concentration, *RDW* red blood Cell distribution width. (b) White blood cell count and analysis. The percentage (%) and absolute number (#) of white blood cells were shown. *WBC* white blood cell count, *LYM* lymphocytem, *MON* moncyte, NEU neutrophil, *BAS* basicyte, *EOS* eosinophil. (c) Platelet analysis. *PLT* platelet count, *PDW* platelet volume distribution width, *MPV* mean platelet volume, *P-LCR proportion of large platelets*, *PCT* thrombocytocrit. *p < 0.05 compared with NP group by ANOVA with Tukey’s test post-hoc. n = 7–10 per group

**Fig. 5:**
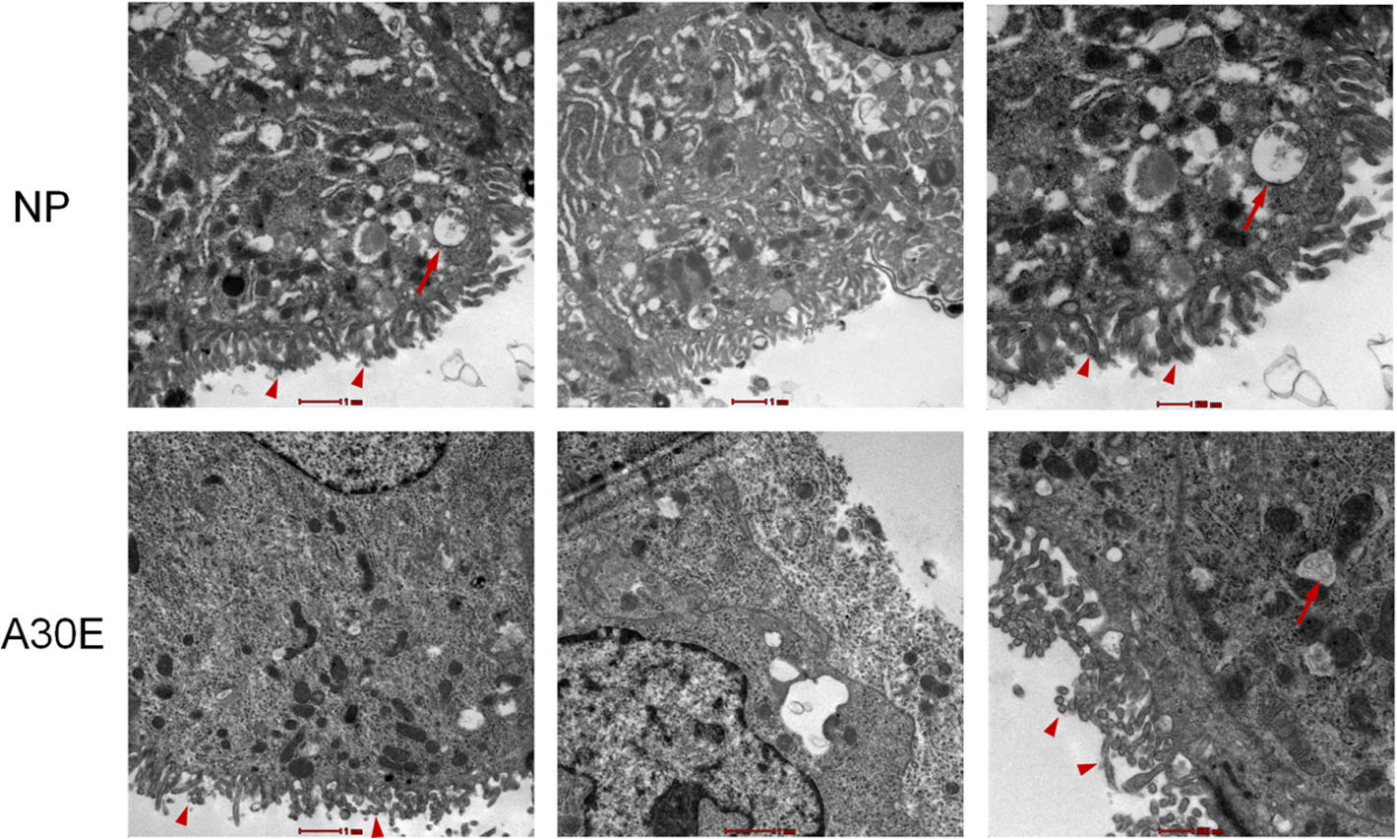
SEM imaging of maternal decidual epithelium. The decidual tissue is from normal pregnant mice of NP group and aborted mice of A30Z group. The diameter of the tissue, which was fixed in 2.5% glutaraldehyde before preparing ultra-thin section, is 1 mm. After uranium and lead staining, the sections were analyzed by Teneo SEM scanning electron microscope (FEI, U.S.A). Arrowheads indicate microvilli. Arrows indicate pinopodes. Representative images of three samples for each group are shown. The scale bars on the two columns on the left are 1μm, and the scale bars  on the one column on the right are 500 nm

We further explored the structural morphology of maternal decidual epithelium. We found that, in NP group, the integral structure of decidual epithelium was densely covered with microvilli, and there were large amounts of pinopodes with distinct structure and abundant mitochondria in decidual cells (Fig. 5). However, for the mice of A30E group, the boundary of the cell membrane was vague, the microvilli were sparse and short, and the density of pinopodes was lower with obscure and shrinkage morphology. Alternatively, abnormal embryonic growth might in turn affect the state of the endometrium to form a deleterious loop (Bauersachs et al., 2009). We checked the circulating progesterone and estrogen concentrations of dams in each group and found that the levels in the animals with aborted fetuses were low, whereas those in dams with healthy fetuses were high, regardless of treatment group (Table 4). For all pregnant mice, the correlation between low estradiol level and abortion state (96.7 ± 5.8%) was significantly higher than that of low estradiol level and A30 exposure (50.0 ± 26.5%); and the correlation between low progesterone level and abortion state (96.7 ± 5.8%) was higher than that of low progesterone level and A30 exposure (50.0 ± 30.0%). (*p* < 0 .01, *n* = 3). This means that changes in maternal sex steroid levels were not the direct results of exposure to A30, but were due to the effects of A30 exposure on fetal viability in susceptible dams (Arck et al., 2007). These results are consistent with the interpretation that A30 directly impacts upon fetal viability causing abortion, rather than indirectly acting on the fetus through adverse effects on maternal health.

**Table 4:** Estrogen serum concentration and level of aborted or non-aborted pregnant mice

Grouping	Mouse number	E2 content (pmol/L)	E2 level definition	P content (nmol/L)	P level definition	Abortion status
NP	NP-1	123.9	H	190.1	H	N
	NP-2	71.6	L	> 190.8	H	N
	NP-3	113.7	H	> 190.8	H	N
	NP-4	111.9	H	> 190.8	H	N
	NP-5	101.5	H	> 190.8	H	N
	NP-6	96.4	L	7.8	L	Y
	NP-7	54.3	L	41.8	L	Y
	NP-8	119.8	H	> 190.6	H	N
	NP-9	116.3	H	> 190.1	H	N
	NP-10	109.5	H	> 190.8	H	N
A30E	A30E-1	67.1	L	3.9	L	Y
	A30E-2	79.4	L	161.8	H	Y
	A30E-3	56.6	L	29.6	L	Y
	A30E-4	123.8	H	> 190.8	H	N
	A30E-5	62.5	L	19	L	Y
	A30E-6	46.8	L	88	L	Y
	A30E-7	124.5	H	> 190.8	H	N
	A30E-8	47.3	L	26.2	L	Y
	A30E-9	111.9	H	> 190.8	H	N
	A30E-10	123.9	H	> 190.8	H	N
A30L	A30L-1	56.1	L	3.9	L	Y
	A30L-2	115.7	H	> 190.8	H	N
	A30L-3	114.7	H	> 190.8	H	N
	A30L-4	62.9	L	15.2	L	Y
	A30L-5	133.1	H	190.1	H	N
	A30L-6	108.4	H	> 190.8	H	N
	A30L-7	121.2	H	190.1	H	N
	A30L-8	126.1	H	> 190.8	H	N
	A30L-9	106.7	H	> 190.8	H	N
	A30L-10	118.3	H	> 190.8	H	N

Pregnant mice in different groups were sacrificed in E16.5 and checked for abortion. In “Abortion status” column, Y means abortion, and N means non-abortion. The serum of each mouse was collected at the same time for the estrogen content assays of estradiol (E2) and progesterone (P). The level of estrogen was defined by comparing with the setting value: E2 concentration above 100 pmol/L is identified as high level (H), lower than 100 pmol/L is identified as low level (L). P concentration above 100 nmol/L is identified as high level (H), lower than 100 nmol/L is identified as low level (L). In the Table, the estrogen concentrations, estrogen level definition and abortion statuses of each mouse were listed correspondingly to show the relationship among abortion, estrogen level change and A30 exposure

### The concentration and distribution of A30 in maternal and fetal tissues

**Fig. 6:**
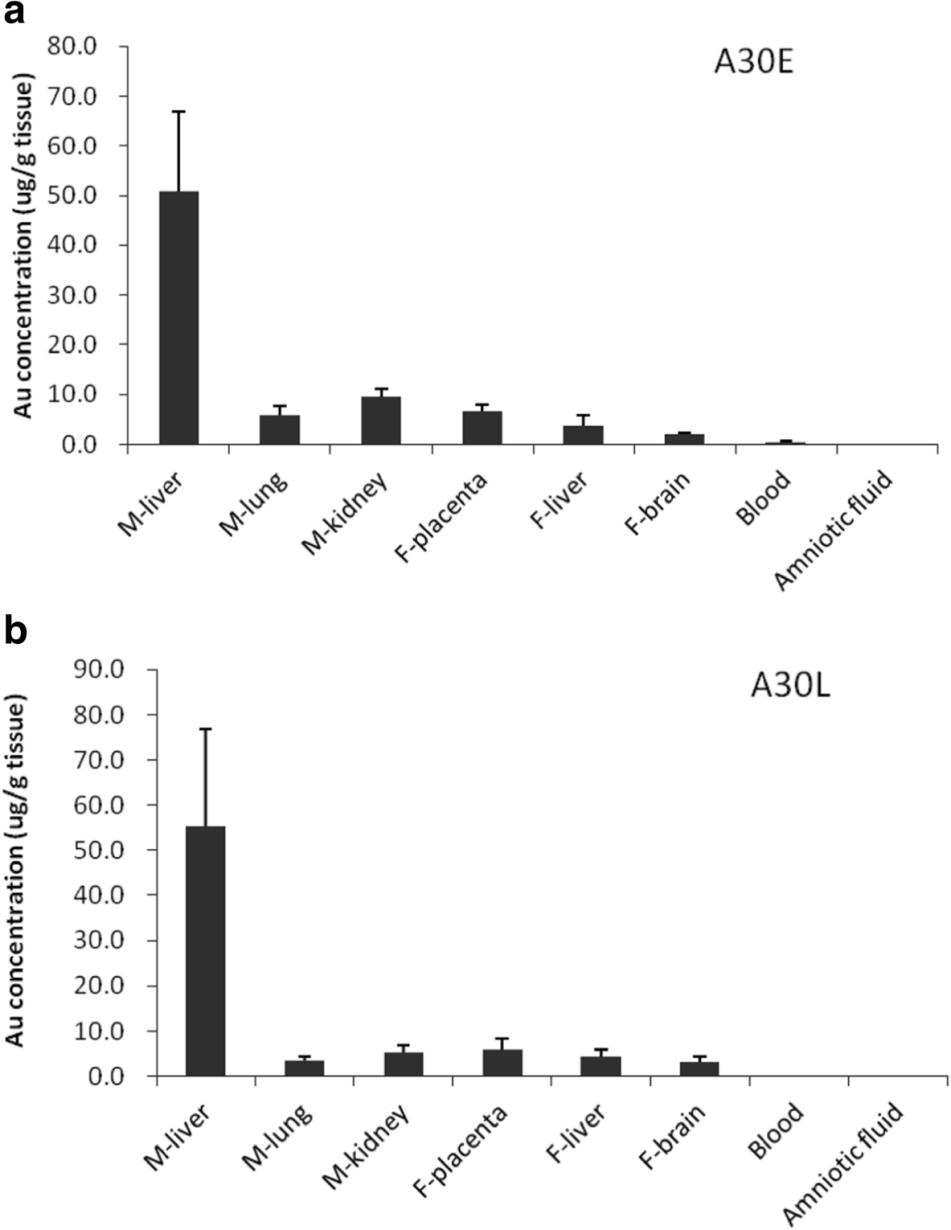
Biological distribution of A30 in pregnant mice and fetuses. **a** Gold concentrations in maternal tissues, fetal tissues, blood and amniotic fluid of A30E group. **b** Gold concentrations in maternal tissues, fetal tissues, blood and amniotic fluid of A30L group. The collected tissues were weighed and digested in aqua fortis, and gold content assays were performed using an ELAN DRC-e ICP-MS instrument (Perkin Elmer, USA). Data are shown as mean ± s .d. (*n* = 6 per group). M-organ, maternal tissue; F-organ; fetal tissue

Inductively coupled plasma mass spectrometry (ICP-MS) quantitative analysis showed that, for the A30E and A30L groups, gold concentrations (shown as μg Au/g organ weight for dam’s or fetal organs) in the maternal liver was 6 to 8 times that of the placenta and other maternal organs (Fig. 6a, b); gold concentrations in maternal blood (shown as μg Au/ml body fluid or blood) was only 0.2 to 1% of that in the liver, indicating that most of the A30 had entered the maternal organs from the circulatory system or excreted out of the body. There was no difference in the concentration of A30 in the maternal organs between the A30E and A30L groups, suggesting that the mild injury of the liver and the kidney function in the A30E group (Fig. 4 c and d) might not be caused by the direct impact from A30, but by an indirect effect of abnormal embryonic development through maternal-fetal interplay. It is worth noting that gold was also detected in fetal tissues (Fig. 6a, b), consistent with the notion that A30 may act directly on embryonic tissue or embryonic cells. The gold concentration in the fetal brain and liver of the A30E group was 2.1 μg Au/g tissue and 3.7 μg Au/g tissue respectively, compared to 3.1 μg Au/g tissue and 4.3 μg Au/g tissue in the A30L group. The amount of gold in amniotic fluid was barely detectable (Fig. 6a, b). These findings suggest that fetal exposure to AuNPs occurs via a trans-placental route rather than via amniotic fluid, resulting in systemic distribution and deposition in the fetal liver and brain.

**Fig. 7:**
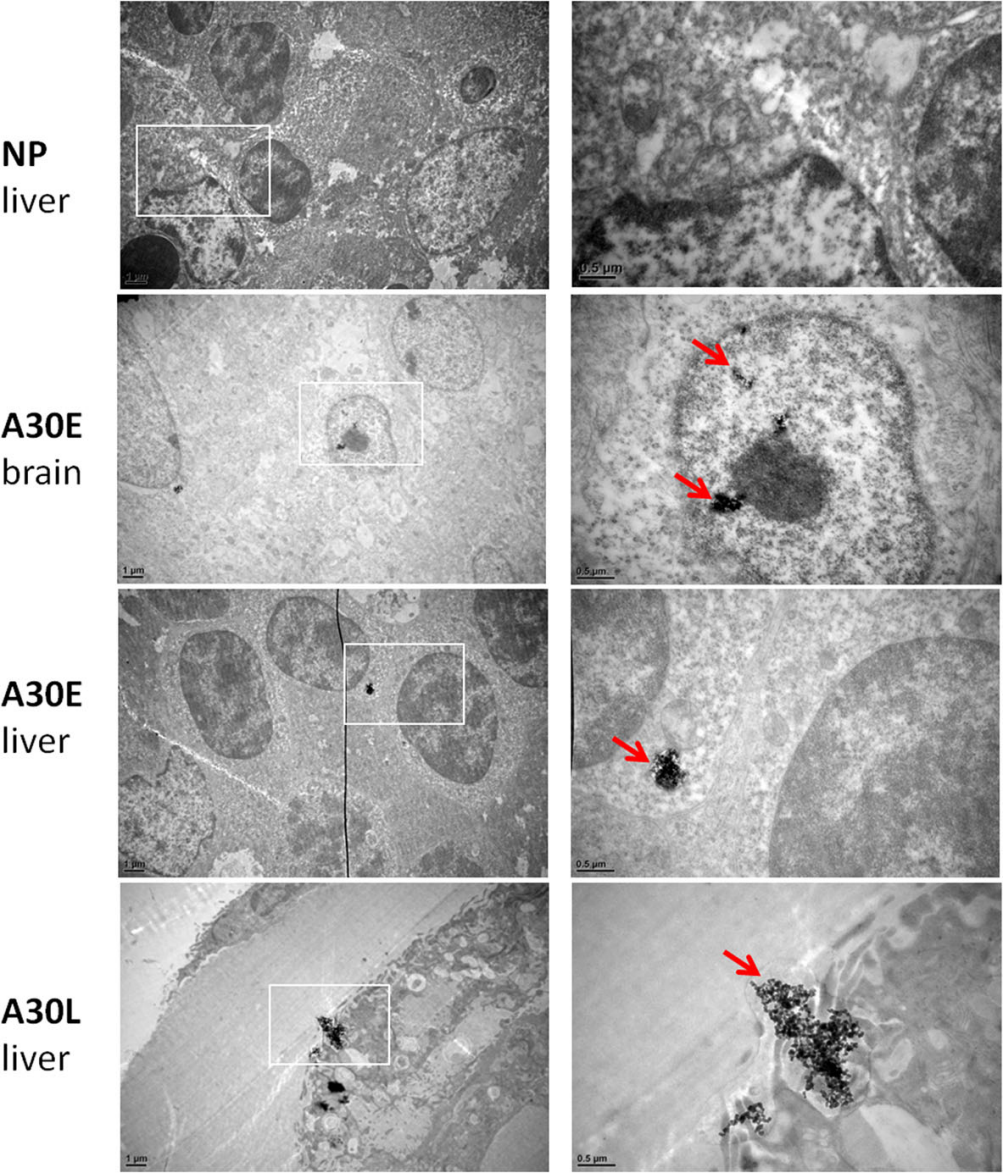
TEM image of E16.5 fetal brain and liver after A30 maternal exposure from E5.5 (A30E) or E11.5 (A30L). Imaging was performed under Tecnai Spirit transmission electron microscope (FEI, U.S.A.). A30 could be found in the fetal liver of the A30E and the A30L groups as well as the fetal brain of the A30E group. The scale bar of left-hand pictures is 1 μm; the amplified images of internal frame area in the left column pictures are shown on right column with scale bar of 0.5 μm. Representative images from three fetal samples of each group are shown here

TEM imaging on E16.5 fetal tissue sections was performed to visualize the presence and physical appearance of A30 in fetal tissues. We found no visible AuNPs in the samples of NP group; however, AuNPs could be observed in the fetal brain of A30E animals and the fetal livers in both A30E and A30L group (Fig. 7). The size of the observed AuNPs was between 25 and 50 nm as expected. A30 could be found in the cytoplasm (Fig. 7, the third line) and even the nucleus (Fig. 7, the second line). As observed at a representative area in the fetal liver section in A30L group (Fig. 7, right column, the fourth line), hundreds of A30 particles formed aggregates, clustered within a vesicular membrane. This A30 cluster appeared to be entering the nucleus through the nuclear membrane. These results approved that A30 in the maternal circulation can cross the placenta, enter major organs of the fetuses, and stayed in the form of clusters or aggregates. Thus, A30 may be enriched in the part of embryos and was likely to have direct impact on the fetal development.

It is worth noting that A30 appears in the form of membranous vesicle-encapsulated clusters or aggregates (Fig. 7), which caused large amounts of AuNPs enriched in a specific small part of an embryo or a few cells of an embryo. Thus, a more serious impact on the early pregnancy embryos with fewer cell numbers compared with the late pregnancy fetuses may happen, i.e. A30 exposure directly affect early embryos development in susceptible animals. For the early embryos with relatively small number of cells, the effects of enriched A30 may be fatal, whereas for more mature fetuses with larger and more developed organs and tissues, sporadic A30 accumulation may have little or no effect on fetal growth. In addition, these oligomeric/cluster enriched A30 may act on the growth and differentiation of some specific embryonic cells without affecting the other cells or tissues that are not exposed to A30 particles. This could explain the “all-or-nothing” characteristics of A30-induced abortion.

### Effect of A30 on differentiation of embryonic stem cells into different germ layer lineages

It is unclear whether there is a direct biological effect of A30 administration on early embryo cells and whether A30 exposure may interfere with the proper differentiation processes of embryonic stem cells. Addressing these questions could contribute to elucidating the cause and mechanism for the loss in fetal viability induced by exposure to A30. In this study, mouse embryonic stem cells R1 (MESC-R1) was used to construct embryoid bodies (EBs), in which MESC-R1 cells autonomously undergo multilineage differentiation after LIF deprivation. By using qPCR, we monitored the expression of multiple lineage markers including Sox1, Sox3, T, Lhx1 and Sox17 in self-renewal MESC-R1 cells and MESC-R1 cell-derived EBs, with or without A30 treatment for 6 days.

**Fig. 8:**
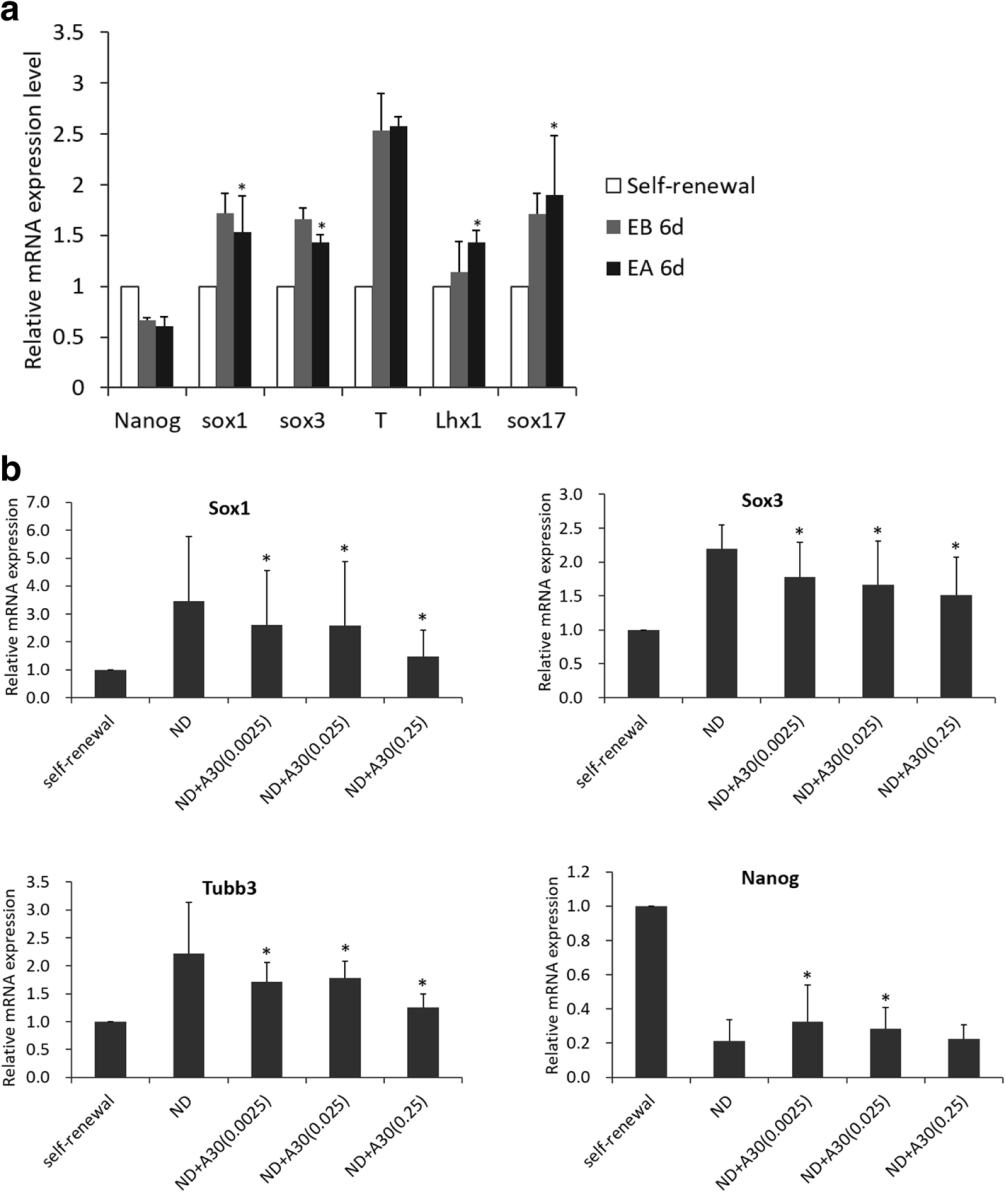
Effect of A30 on differentiation of embryonic stem cells into different germ layer lineages. Nanog, stemness marker; Sox1, Sox3 and Tubb3, ectodermal differentiation markers; Lhx1, mesodermal differentiation marker; Sox17, endodermal differentiation marker; T, mesendodermal differentiation marker. **a** mRNA expression analysis of ectodermal, mesodermal and endodermal differentiation markers in MESC-R1 derived embryoid body (EB) upon the A30 treatment for 6 days after EB formation. Self-renewal, undifferentiated MESC-R1 cells; EB, embryoid body-differentiated MESC-R1 cells; EA, embryoid body differentiated MESC-R1 cells treated with 0.25 μg/ml of A30. **b** mRNA expression analysis of ectodermal differentiation markers in neuroectodermal differentiated MESC-R1 cells upon the A30 treatment at different concentrations for 5 days. Self-renewal, undifferentiated MESC-R1; ND, neuroectodermal differentiated MESC-R1. A30 concentration umit is μg/ml. The symbol * represents *p* < 0.01 compared with EB group (**a**) or ND group (**b**) by ANOVA with Tukey’s test post-hoc for the corresponding mRNA expression (*n* = 3)

The mRNA expression levels of the mesodermal marker gene Lhx1 and endodermal marker gene Sox17 in A30-treated EBs were significantly higher than those in non-A30-treated EBs, whereas the mRNA expression of mesendodermal marker gene T remained unchanged (Fig. 8a). Interestingly, the ectodermal marker genes Sox1 and Sox3 both exhibited significant downregulation of expression in A30-treated EBs compared with non-A30-treated EBs. These results suggested that there might be a differential pattern of regulation for ectodermal differentiation in response to A30 treatment. MESC-R1 differentiation towards a neuroectodermal phenotype was further induced by nerve cell growth additive N2 and B27 supplement (Gibco, U.S.A.). The expression of a series of neuroectodermal differentiation marker genes was detected by qPCR 5 days after neuroectodermal induction. A30 treatment induced a significant concentration-dependent downregulation of ectodermal markers Sox1, Sox3 and Tubb3 expression compared with non-A30-treated cells (Fig. 8b). These results suggest that exposure to A30 greatly inhibited neuroectodermal differentiation of mouse embryonic stem cells during germ formation, which might contribute to developmental abnormality of murine embryos. In a previous report, the exposure to 10 nm and 30 nm NPs inhibited the differentiation of embryonic stem cells into contracting cardiomyocytes (Park et al., 2009). Fluorescent nanoparticles were reported to influence embryonic stem cell differentiation by disruption of cytoskeletal development (Tran et al., 2007). Polyethylene NPs (PE-NPs) penetrated deep into a human embryonic stem cell-derived 3-D structures and downregulated neuronal precursor genes such as NEUROD1 and ASCL1, resulting in severe impairments of the nervous system in mice (Hoelting et al., 2013). The development of the nervous system and the myocardium both occur during the early stage of embryonic development.

The present study demonstrates that A30 in maternal circulation could cross immature placenta into the embryos and reach the fetal brain and other tissues. Therefore, exposure to A30 during early pregnancy could act directly on embryonic stem cells. We found that three consecutive exposures to A30 during early - but not late - pregnancy leads to significant adverse effects on fetal viability. A30 exposure in early pregnancy may interfere with multiple lineage differentiation of ES cells, specifically inhibiting the ectoderm differentiation and nervous system development. After that window of time during early pregnancy, probably due to the completion of differentiation of the germ layer, the effects of A30 on embryonic development were much less significant. This phenomenon fits well with the “all-or-nothing” pattern, which is commonly seen in neurodevelopmental disorders. The findings from our MESC-R1 studies provide some direct evidence and explanation for the abnormality of fetal development via blocked ectodermal differentiation. A30 exposure might also indirectly suppress fetal development through alterations in the maternal physiological function (Fig. 4, Tables 2 and 3). While we have no empirical evidence on the relative contributions of potential direct and indirect effects, it is likely that the direct impact on fetal development plays the more important role in the process.

## Conclusions

We have explored for the first time the effects of repeated maternal exposure of 30 nm AuNPs on fetal development and viability. We showed that exposure to A30 during early pregnancy, which corresponds to the first trimester of human pregnancy, resulted in a significant increase in the rate of abortions and stillbirths, but does not lead to obvious developmental deficits in surviving offspring. The developmental damage caused by A30 exposure followed an “all-or-nothing” pattern. Mild liver and kidney dysfunction and immune activation were observed in dams exposed to A30 in early pregnancy, which might exert an indirect influence on fetal development. Furthermore, we found that A30 accumulates in clusters in fetal tissues following early pregnancy exposure. We showed that A30 inhibits neuroectodermal, but not mesodermal and endodermal, differentiation of embryonic stem cells during germ formation, which may be an important contributor to aberrant fetal development during early pregnancy. These findings offer important biosafety clues to help design preventive or therapeutic applications to maintain maternal and fetal health during pregnancy under exposure of nanoparticles.

## Additional file

Additional file 1: **Figure S1.** Synthesis and characterization of gold nanoparticles A30. (A) Schematic illustration of an A30 nanoparticle. (B) A30 suspension in ddH_2_O. (C) TEM image showing the morphology of A30. **Figure S2.** The representative images of aborted and non-aborted uteri after A30 administration. (A) non-aborted uterus. (B) aborted uterus and (C, D) abnormal development uterus. Arrows indicate abortion site with no content, arrowheads indicate stillbirth with turbid red or black contents in the uterus “bead”. Scale bar is 1 cm. **Figure S3.** Ammonium sulfide staining of the uterus without embryo. The left tissue comes from the A30E group, and the right tissue comes from the A30L group. Arrow shows the implantation sites in the uterus without embryo after ammonium sulfide staining. **Figure S4.** Morphology of the placentas and fetuses in non-aborted mice of NP and A30-exposed groups. The left column is the whole uterus of E16.5 mice, including fetuses. The right column is the dissected fetus and placenta. (RTF 16451 kb)

## Abbreviations

A30: 30 nm polyethylene glycol (PEG)-coated AuNPs
A30E: Exposure to A30 during early pregnancy
A30L: Exposure to A30 during late pregnancy
AuNPs: Gold nanoparticles
ICP-MS: Inductively coupled plasma mass spectrometry
MESC-R1: Murine embryonic stem cell R1
SEM: Scanning electron microscopy
TEM: Transmission electron microscopy
