# Supplementary material for: Murine exposure to gold nanoparticles during early pregnancy promotes abortion by inhibiting ectodermal differentiation
Source: Mol Med. 2018 Dec 3;24:62. doi: 10.1186/s10020-018-0061-2 (PMC6276159; doi:10.1186/s10020-018-0061-2)
Supplement: Supplementary file 1 — Figure S1. Synthesis and characterization of gold nanoparticles A30. (A) Schematic illustration of an A30 nanoparticle. (B) A30 suspension in ddH2O. (C) TEM image showing the morphology of A30. Figure S2. The representative images of aborted and non-aborted uteri after A30 administration. (A) non-aborted uterus. (B) aborted uterus and (C, D) abnormal development uterus. Arrows indicate abortion site with no content, arrowheads indicate stillbirth with turbid red or black contents in the uterus “bead”. Scale bar is 1 cm. Figure S3. Ammonium sulfide staining of the uterus without embryo. The left tissue comes from the A30E group, and the right tissue comes from the A30L group. Arrow shows the implantation sites in the uterus without embryo after ammonium sulfide staining. Figure S4. Morphology of the placentas and fetuses in non-aborted mice of NP and A30-exposed groups. The left column is the whole uterus of E16.5 mice, including fetuses. The right column is the dissected fetus and placenta. (RTF 16451 kb) [file 10020_2018_61_MOESM1_ESM.rtf]

Additional file 1

Figure S1. Synthesis and characterization of gold nanoparticles A30.


Figure S2. The representative images of aborted and non-aborted uterus after A30 administration.


Figure S3. Ammonium sulfide staining of the uterus without embryo.


Figure S4. Morphology of the placentas and fetuses in non-aborted mice of NP and A30-exposed groups.
